# Outcomes Following Iliac Vein Stenting for Non-Thrombotic Iliac Vein Lesions—A Narrative Review Based on Large Sample Studies

**DOI:** 10.3390/jfb16120427

**Published:** 2025-11-22

**Authors:** Arjun Jayaraj

**Affiliations:** The RANE Center for Venous & Lymphatic Diseases, St. Dominic Hospital, Jackson, MS 39216, USA; jayaraj.arjun2015@gmail.com; Tel.: +1-(601)-939-4230

**Keywords:** iliac compression syndrome, iliac vein compression, iliac vein stenosis, May Thurner syndrome, non-thrombotic iliac vein lesion, iliac vein stenting, venous stenting

## Abstract

*Objective:* May–Thurner syndrome typically refers to symptoms and signs arising from the compression of the left common iliac vein by the right common iliac artery. However, such clinical manifestations can occur in the setting of compression of the right common iliac vein and/or either external iliac vein. Given this scenario, the more appropriate term for the condition would be non-thrombotic iliac vein lesion(s) [NIVL]. The goal of this review of large sample size studies is to evaluate outcomes following stenting for chronic iliofemoral venous obstruction (CIVO) due to NIVL, including clinical, quality-of-life, and stent-related outcomes. Additionally, where evidence gaps or controversies exist, expert opinion has been offered for guidance. *Methods:* A review of the literature was undertaken to determine the role of stenting for NIVL. Appropriate search terms were used to search PubMed, Cochrane Central Register of Controlled Trials, and EMBASE. Studies were only included if they had a sample size of at least 100 limbs that underwent stenting for NIVL and had at least 12 months of follow-up. Additionally, every study needed to have at least one metric of objective clinical evaluation [Venous clinical severity score (VCSS)] and/or a quality-of-life (QoL) measure (generic or venous disease-specific). *Results:* A total of six studies met the eligibility criteria and included 1404 limbs that underwent stenting for NIVL. All except three studies had a combination of PTS and NIVL limbs, with all six studies having at least 100 limbs that underwent stenting for NIVL. Follow-up varied from 12 to 50 months post-stenting. Improvements in VCSS and quality-of-life measures were noted post-stenting. Additional outcome measures, like grade of swelling or visual analog scale pain score, when utilized, also demonstrated improvement. Recurrence-free ulcer healing rates of 63% to 82% were observed. Good long-term stent primary stent patencies (74–98%) were also reported, irrespective of stent type. *Conclusions:* This review notes that good outcomes can be expected following stenting for CIVO due to NIVL. Gaps, however, exist with regard to patient selection, peri/post-procedural antithrombotic strategies, and long-term follow-up in this context. A CEAP clinical class-based algorithm is provided to help with patient selection in addition to guidance on antithrombotic therapy and follow-up. Further study of these areas is merited.

## 1. Introduction

Stenting has become the first line of therapy for patients presenting with symptomatic chronic iliofemoral venous obstruction (CIVO) not responding to conservative therapy [1,2,3,4,5,6,7,8,9,10]. Such obstruction can be due to a non-thrombotic iliac vein lesion (NIVL), post-thrombotic obstruction or, at times, a combination of the two. While stenting in the early years was performed through the use of non-dedicated stents, the last decade has witnessed the arrival and increasing use of dedicated venous stents [11,12,13,14]. The goal of stenting is to mitigate the resultant venous hypertension by correcting such obstruction. While the technique of stenting is essentially the same for both types of lesions, the stent patencies are, however, different, with better patencies noted for NIVL [3,15,16,17,18,19,20]. In addition to stent patencies, clinical and quality-of-life outcomes must also be considered. The latter are particularly important given that in venous disease, for the most part, unlike arterial disease, intervention is to improve the quality of life and not to save the limb or life. The patency of a stent does not necessarily translate into clinical or quality-of-life improvement. This review primarily focuses on the outcomes following stenting for symptomatic CIVO due to NIVL. While doing so, it also explores the indications, technique, and post-procedural care in this context. Given the controversies surrounding these aspects, this review has sought to provide clarification, incorporating evidence where available and offering expert opinion where such evidence is lacking.

## 2. Methods

### 2.1. Search Strategy

Search terms including May–Thurner syndrome, iliac compression syndrome, iliac vein compression, non-thrombotic iliac vein lesion, iliac vein stenosis, deep venous obstruction, venous stenting, iliac vein stenting, endovenous stenting, chronic venous insufficiency, and venous leg ulcer were used to query PubMed, Cochrane Central Register of Controlled Trials (CENTRAL), and Embase databases. These searches were restricted to manuscripts (not abstracts) of studies published in English from 2000 to 2025. References of these studies were then reviewed for additional studies, which were then searched for manually.

### 2.2. Eligibility Criteria

Studies were only included if they had a sample size of at least 100 limbs that underwent stenting for NIVL/May–Thurner syndrome and had at least 12 months of follow-up. Additionally, every study needed to have at least one metric of objective clinical evaluation [Venous clinical severity score (VCSS)] and/or a quality-of-life measure, either disease-specific (chronic venous insufficiency quality-of-life questionnaire [CIVIQ-20] or Venous Insufficiency Epidemiological and Economic Study Quality of Life [VEINES—QOL]) or generic (e.g., SF-36 or EuroQol). Studies that did not have either a clinical outcome or a quality-of-life measure were excluded. Studies where patients underwent stenting exclusively for post-thrombotic syndrome, chronic total occlusive (CTO) lesions, or in the acute setting (deep venous thrombosis) were also excluded.

## 3. Results

A total of six studies met the eligibility criteria and included 1404 limbs that underwent stenting for NIVL [15,16,21,22,23,24]. All except three studies had a combination of PTS and NIVL limbs, with all six studies having at least 100 limbs that underwent stenting for NIVL. Follow-up varied from 12 to 50 months post-stenting. The characteristics of the six studies are considered in Table 1.

### 3.1. Clinical Outcomes

#### 3.1.1. Grade of Swelling

Three studies reported on impact of stenting on the grade of swelling (GOS). Neglen et al. [15] and Jayaraj et al. [16] assessed swelling as grade 0—absent; grade 1—pitting, not obvious; grade 2—visible ankle swelling; grade 3—gross swelling involving the leg up to knee; and grade 4—gross swelling involving the entire leg including the thigh. Grade of swelling was characterized by Ye et al. [21] as none (0), evening edema in the ankle only (1), afternoon edema above the ankle (2), or morning edema above the ankle requiring activity change (3). After stenting, while Neglen et al. [15] reported an improvement in the grade of swelling from 1.7 to 0.8 (*p* < 0.0001), Ye et al. [21] noted an improvement in the grade from 1.7 to 0.6 (*p* < 0.01), and Jayaraj et al. [16] observed an improvement in the grade of swelling from 3 to 1 (*p* < 0.0001). At 5 years, Neglen and colleagues [15] noted that 32% of limbs remained completely free of swelling. The prevalence of severe swelling decreased from 36% to 18% with post-thrombotic limbs appearing to be more frequently free from swelling than NIVL limbs (39% vs. 24% *p* = 0.004). In the study by Ye et al. [21] the cumulative relief of edema was 79.2% (156/175 limbs).

#### 3.1.2. Visual Analog Scale Pain Score

Here again, the same three studies that had evaluated GOS also evaluated the VAS pain score. While Neglen and colleagues [15] reported a VAS pain score improvement from 3.7 to 0.8 (*p* < 0.0001), Ye et al. [21] demonstrated a VAS pain score improvement from 4.3 to 0.4 (*p* < 0.01) after stenting and Jayaraj et al. [16] reported an improvement in the VAS pain score from 5 to 2 (*p* < 0.0001) after stenting. Neglen et al. [15] noted that at 5 years 62% of limbs remained completely free of pain. The prevalence of limbs with severe pain decreased from 41% to 11%. There was no significant difference in the prevalence of complete relief of pain at 5 years between the post-thrombotic and NIVL limbs—59% vs. 65% (*p* = 0.7). Yang et al. [22] noted a cumulative pain relief of 71.1% in NIVL limbs that underwent endovenous laser ablation (EVLA) with stenting while only 43.5% had such relief in NIVL limbs that underwent EVLA alone (*p* = 0.03).

#### 3.1.3. Combined Pain and Swelling

Jayaraj and colleagues [16] noted a complete relief of pain and/or swelling in 60% of limbs, partial relief of pain and/or swelling in 25% and no relief in the remaining (15%) limbs. Importantly, no worsening was noted in the study population throughout the follow-up period [16].

#### 3.1.4. Ulcer Healing

While Neglen et al. noted a recurrence-free ulcer healing rate of 63% over 5 years of follow up, Ye and colleagues [21] reported a 48-month recurrence-free ulcer healing rate of 82.3% and Jayaraj and colleagues observed a recurrence-free ulcer healing rate of 66%. Yang and counterparts [22] found that EVLA with stenting improved the recurrence-free ulcer healing rate to 82.8% compared to 68.8% with EVLA alone. Hong and colleagues [23] noted an ulcer healing rate of 91% (Venastent, Tianhong, China) vs. 83% (Zilver vena, Cook Medical, Bloomington, IN, USA) in NIVL limbs that underwent stenting without a significant difference (*p* = 0.9) between the two groups.

#### 3.1.5. Venous Clinical Severity Score (VCSS)

Four studies evaluated and reported VCSS before and after stenting. While Jayaraj et al. [16] found a VCSS improvement from 6 to 4 (*p* < 0001) over a median follow-up of 26 months, Yang et al. [22] found that EVLA with the addition of stenting resulted in a lower mean VCSS of 8.3 compared to 11.7 with EVLA alone (*p* = 0.01). Hong et al. [23] noted a significant improvement in revised VCSS at 12 months (4.4 vs. 5), irrespective of the stent type used. Ortega et al. [24] reported a mean VCSS improvement from 6.9 to 1.4 for NIVL limbs post-stenting.

### 3.2. Quality-of-Life Outcomes

Quality of life was assessed in three studies, with Neglen and colleagues [15] reporting a statistically significant improvement in the CIVIQ score over a mean follow-up of 22 months (*p* < 0.001). Ye et al. [21] also reported a statistically significant improvement (*p* < 0.001) while Jayaraj and colleagues [16] noted a 24-point improvement (*p* < 0.0001) over a median follow-up of 26 months.

### 3.3. Stent-Related Outcomes

Of the six studies, five provided patency data. Of these, three use patency as defined by the societal guidelines [25,26]. The other two studies, Ye et al. and Hong et al., did not report on patency definitions. The study by Yang et al. did not report patency. At 72 months, Neglen et al. [15] noted a primary and primary assisted cumulative patency rates of 79%, and 100% in 518 limbs with NIVL. There were no stent occlusions in the NIVL cohort and severe (>50%) instent restenosis (ISR) occurred in 1% of limbs [15]. In the 224 NIVL limbs that underwent stenting, Ye et al. [21] reported primary and primary assisted cumulative patency rates of 98.7% and 100%, respectively, over a follow-up of 4 years. In the study by Jayaraj et al. [16] where 104/545 limbs had NIVL and underwent stenting, the primary patency at 60 months was 74%, and primary assisted patency was 100%. Hong and colleagues [23] noted primary patencies of 100% (Venastent) and 98.4% (Cook Zilver) at 12 months in the 246 limbs that underwent stenting for NIVL. Ortega et al. [24] reported primary patency of 98.2% at 3 years and primary assisted patency of 100%. Both reinterventions were for instent restenosis. Across all studies the primary patency for NIVL is quite robust with reinterventions occurring for ISR. No stent occlusions were reported for NIVL limbs in any of the studies.

### 3.4. Complications

Neglen et al. [15] reported access site complications that included one retroperitoneal bleed, one femoral artery injury, three femoral artery pseudoaneurysms (treated with ultrasound guided thrombin injection [UGTI]) and one AV fistula (closed spontaneously). Contralateral iliac deep vein thrombosis (DVT) was noted in four limbs, while access site DVT was noted in two, and calf DVT in one. No stent fractures were observed. There was no 30-day mortality [15]. Ye et al. noted stent migration in three limbs that required placement of additional stents to anchor the first (≈1.3%) [21]. Access site hematoma was noted in two. There was no 30-day mortality [21]. Jayaraj et al. reported procedure-related hemorrhage requiring transfusion of blood products in five patients, and pseudoaneurysms in four limbs (three required UGTI and one closed spontaneously) [16]. An arteriovenous fistula was observed in one limb and managed conservatively. There was one instance of contralateral iliac vein thrombosis. Additionally, there was a superficial femoral artery injury that required the placement of a covered stent. No deaths were reported [16]. In the study by Yang et al., DVT, pulmonary embolism (PE), or deaths were not observed in either group [22]. Hong et al. reported one instance of ipsilateral DVT and PE that required lysis [23]. There were seven instances of hemorrhage related to oral anticoagulants. These complications did not vary between the two groups [23]. Ortega et al. reported vein rupture in one patient and contralateral thrombosis in four patients [24].

## 4. Discussion

Over the last two decades, stenting has replaced open surgery as the treatment modality for patients with symptomatic chronic iliofemoral venous obstruction who have failed conservative therapy. Randomized trials have noted the benefit of such stenting versus conservative therapy [27,28]. However, with this increased utilization, the number of studies conducted to evaluate stenting in this setting has also increased. Unfortunately, the focus in many of these studies has been to assess stent patency as opposed to clinical or quality-of-life improvements which should be the primary outcome measures in venous interventions, as such interventions for most part, unlike arterial interventions, are not performed to save a limb or life. This review has attempted to define the role of stenting in this context by focusing on large studies that have such outcome measures following stenting for NIVL. Gaps and controversies do, however, exist surrounding the indications, technique, and post-procedural care in this setting [29]. This review and, in particular, the discussion section (Section 4) below have sought to provide clarification, incorporating evidence where available and offering expert opinion where such evidence is lacking. Relevant references have been provided where they exist. Their absence should be interpreted as expert opinion based on the author’s extensive experience on the subject.

### 4.1. Patient Selection for Iliofemoral Venous Stenting

**Figure 1:**
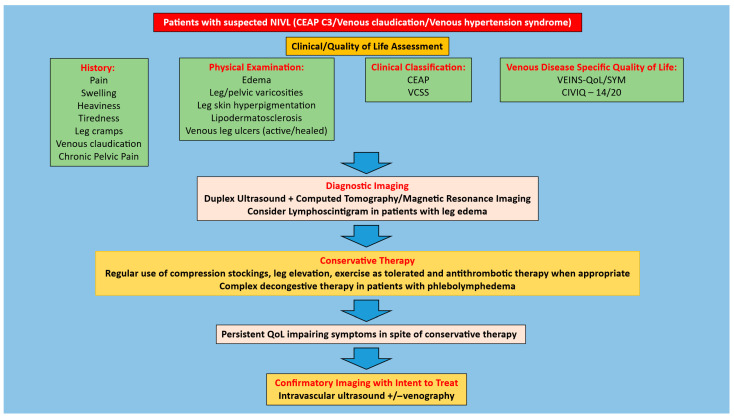
Algorithm for management of patients with CEAP clinical class 3, venous claudication, or venous hypertension syndrome and suspected NIVL. Adapted from *Jayaraj A, Rossi FH, Lurie F, Muck P. Diagnosis of chronic iliac venous obstruction. J Vasc Surg Venous Lymphat Disord. 2024 Jul;12(4):101744*.

**Figure 2:**
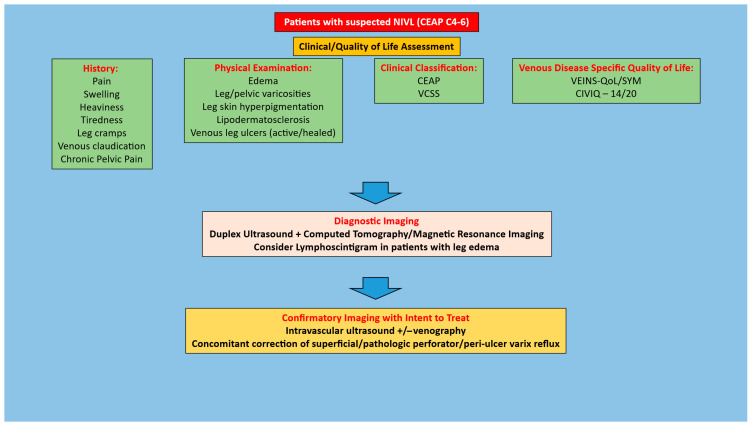
Algorithm for management of patients with CEAP clinical class 4–6 and suspected NIVL. Adapted from *Jayaraj A, Rossi FH, Lurie F, Muck P. Diagnosis of chronic iliac venous obstruction. J Vasc Surg Venous Lymphat Disord. 2024 Jul;12(4):101744*.

It is important to start with a detailed history and physical focusing on eliciting signs/symptoms of chronic venous insufficiency that would be suggestive of chronic iliofemoral venous obstruction. Such clinical manifestations can include pain, swelling, heaviness, tiredness, tightness, leg cramps, venous claudication, hyperpigmentation, lipodermatosclerosis, and/or venous leg ulcers (active or healed). In patients with a high degree of suspicion for CIVO, the next step is to obtain diagnostic imaging studies. This would include a duplex ultrasound of the lower extremity and pelvis to assess for an obstruction and/or reflux in addition to cross-sectional imaging. The latter is typically a computed tomography venogram, although magnetic resonance imaging can also be used [30,31]. A lymphoscintigram should also be performed in patients with leg edema [32]. In patients with quality-of-life impairing clinical manifestations and diagnostic imaging suggestive of NIVL, treatment is based on their Clinical-Etiology-Anatomy-Pathophysiology (CEAP) clinical class. In patients with CEAP C3 or lower (including venous claudication or venous hypertension syndrome), conservative therapy in the form of regular use of compression stockings, leg elevation, exercise as tolerated, weight loss when indicated, and complex decongestive therapy in patients with edema should be pursued as the initial treatment. In patients who fail such conservative therapy, as well in patients with CEAP C4–6 disease (given end organ damage—skin/soft tissue damage), IVUS interrogation for confirmation of diagnosis and subsequent stenting once the diagnosis is confirmed should be pursued. In NIVL limbs with CEAP C6 disease, concomitant correction of any superficial and/or peri ulcer pathologic perforator/varicose vein reflux should also be pursued. Algorithms for the management of symptomatic patients with suspected NIVL are provided in Figure 1 and Figure 2. There is no role for prophylactic stenting in the absence of symptoms or stenting in the setting of mild symptoms without quality-of-life impairment. NIVL are quite common in the general population [33,34,35,36] and any treatment should be based purely on quality-of-life impairing symptoms or end organ (skin/soft tissue) damage.

### 4.2. Stenting for NIVL

Access for stenting is typically obtained in the mid-thigh femoral vein using ultrasound guidance, and an 11Fr sheath is placed. Such access enables the stent extension below the inguinal ligament if necessary, something that is not possible if the access is obtained in the common femoral vein given the length of the sheath (usually about 10 cm). The next step is to confirm the diagnosis of chronic iliofemoral venous obstruction. This is achieved through a 0.035 IVUS catheter (Visions PV 0.035 digital IVUS catheter; Philips, Amsterdam, the Netherlands). It is important to bear in mind that the confirmation of a diagnosis of NIVL should be based on IVUS and not venography alone [31,37,38,39,40]. Additionally, such IVUS criteria should be through the use of normal minimal luminal areas as opposed to comparative criteria (ipsilateral ‘normal’ vein or contralateral ‘normal’ vein) given the presence of multifocal and bilateral disease. The use of 50% stenosis to confirm a diagnosis should also be avoided, as this concept is extrapolated from arterial literature and has no basis in venous disease [41,42,43]. In veins, venous hypertension arising from obstructive lesions tends to be continuous and nonlinear, with a variety of mechanisms contributing to the development of such hypertension. Such mechanisms include the absence of collateral venous outflow, absence of a compensatory increase in lymphatic flow, impaired calf pump function, loss of vein wall compliance, the existence of venous reflux (superficial and/or deep), right heart dysfunction, and impaired respiratory status. The development of symptoms/signs of chronic venous insufficiency in a patient with NIVL is indicative of the absence or failure of compensatory mechanisms and the need to correct the obstruction. Normal luminal area cut-offs used to diagnose NIVL are 125 mm^2^, 150 mm^2^, and 200 m^2^ in the common femoral, external iliac, and common iliac veins, respectively [44]. Any reduction in the luminal areas below these cut-offs in a patient meeting the aforementioned criteria merits stenting. The principles of stenting call for coverage of all areas of disease (NIVL can often be multifocal), with stenting extending from an area of good inflow into an area of good outflow. Stent sizes are determined based on the IVUS inflow channel luminal area [45]. Both predilation and post-dilation are important with angioplasty balloon sizes dictated by the size of the stent to be used. Following post-dilation, a completion IVUS interrogation is performed to ensure the adequacy of stenting and a venogram to ascertain final flow dynamics.

### 4.3. Discharge and Follow-Up Post-Stenting for NIVL

Patients are usually discharged the same day unless the pain or medical comorbidities require overnight observation. With regard to antithrombotic therapy in NIVL patients, anticoagulation is typically continued in patients who were already on it preoperatively, patients with thrombophilia, patients with a history of an unprovoked venous thromboembolic event, patients on hormonal therapy, and patients with early severe ISR on post-procedure duplex ultrasound (DUS). When anticoagulation is started post-stenting, a direct oral anticoagulant (DOAC) is typically used and usually continued for 3–6 months based on the patient’s clinical status and DUS results on follow-up. Aspirin 81 mg daily is started and continued lifelong as long as no contraindications are present. From a post-procedure follow-up standpoint, a DUS is performed before discharge on the day of the intervention, with additional DUS and clinic visits at 3 weeks, 3 months, 6 months, and 12 months and annually thereafter, as long as the patients remain asymptomatic without evidence of a stent malfunction. A closer follow-up is indicated if there is concern for clinical recurrence and/or a stent malfunction. The technique of stenting, peri/post-operative care and follow-up have been described in prior publications [16,45].

### 4.4. Clinical Improvement Following Stenting

There is clinical improvement across all six studies in the review. Improvement in the grade of swelling was noted in the studies by Neglen et al. [15], Ye et al. [21], and Jayaraj et al. [16]. The same three studies, in addition to the one by Yang et al. [22], note improvement in pain as assessed by the VAS pain scale. However, a complete relief of pain occurs in a greater proportion of limbs (~60%) than complete relief of swelling (~30%). Improvement in VCSS ranged from 2 to 8.3 post-stenting. Long-term recurrence-free ulcer healing ranged from 63% to 82.3% in the studies. Thus, while there is significant improvement in clinical metrics, a sizeable number of patients have persistent or residual symptoms. Persistence of swelling post-stenting is likely to be related to phlebolymphedema, and such patients will benefit from measures focused on treating the lymphedema component, including complex decongestive therapy (CDT). Axial deep venous reflux is another possibility in this situation and patients with this problem would benefit from the correction of such reflux, either through open or endovenous means if stenting and CDT do not provide adequate relief. It should be borne in mind that in patients with combined deep venous obstruction and reflux, the obstruction should be corrected first since only a small percentage of patients continue to have significant deep reflux contributing to persistent symptoms following the correction of such obstruction [16,46,47]. From a pain standpoint, persistence of quality-of-life impairing leg pain could be related to permanent alteration in the venous hemodynamics/vein wall compliance resulting in chronic compartment syndrome. This typically involves the posterior superficial compartment of the leg, and the measurement of compartment pressures in the supine, upright, and post-exercise modes (based on symptom onset) helps confirm such a diagnosis. Pursuance of posterior superficial compartment fasciotomy and fasciectomy usually provides the necessary symptom relief in patients with elevated compartment pressures (>15 mm Hg) [48]. A correction of the venous outflow obstruction should precede such an intervention, given the low overall incidence of chronic compartment syndrome and the much higher prevalence of iliofemoral venous obstruction.

### 4.5. Quality-of-Life Improvement Post-Stenting

Of the three studies that evaluated quality of life, all three utilized the CIVIQ-20 questionnaire. All the studies demonstrated an improvement in both the global and individual domain scores post-stenting that was statistically significant. This represents an important finding as in most situations, the main reason for intervention in venous disease, as noted previously, is to improve the patient’s quality of life [49].

### 4.6. Stent-Related Outcomes

Excellent stent patencies have been noted over the long term. The predominant reason for reintervention, when needed, was primarily for instent restenosis. No stent occlusions were noted in any of the studies, demonstrative of the finding that this is primarily a problem in limbs undergoing stenting for post-thrombotic obstruction. No other stent-related complications, including migration, were noted in the studies. While stent migration has been noted in the literature, this is a rare occurrence occurring primarily when smaller (<14 mm) and shorter (<60 mm length) stents are used [50]. It is important for interventionalists to bear this in mind. Also, while the type of stent used does not appear to impact outcomes for NIVL [51], from a technical standpoint it is better to use a composite Wallstent—Z stent combination as opposed to Wallstent alone given the increased risk for the development of contralateral symptoms and thrombosis [16,52]. This is less of an issue with the use of dedicated venous stents [51,53]. As with the initial stent placement, reintervention for NIVL should be for quality-of-life impairing recurrent symptoms as opposed to a set degree of instent restenosis or stent compression [54].

### 4.7. Study Heterogeneity

There is a heterogeneity among the six studies, primarily in terms of the diagnostic modalities used to confirm the diagnosis and guide stenting. Four studies are retrospective cohort studies without a comparator group. One retrospective cohort study compares stenting to stenting with endovenous laser ablation of superficial/perforator veins and additional therapy for varicose veins. The RCT has sought to compare two stent types. When the risk of bias tool was applied to this study, it was noted that the randomization had a low risk of bias, while the other domains had some concerns, with an overall risk of bias for the RCT as ‘some concerns’.

## 5. Conclusions

Good outcomes, clinical, quality-of-life, and stent-related, can be expected following stenting for NIVL. Gaps, however, exist with regard to patient selection, peri/post-procedural antithrombotic strategies, and long-term follow-up in this context. From an indications standpoint, the use of a CEAP-based treatment algorithm can be helpful. Based on this, in patients with CEAP C3 disease, venous claudication, or venous hypertension syndrome, stenting should be reserved for those who have QoL impairing clinical manifestations and have failed conservative measures. However, in patients with CEAP C4–6, given the presence of end-organ damage (hyperpigmentation, lipodermatosclerosis, and/or venous leg ulcers), stenting should be pursued as the initial treatment. IVUS interrogation should be used to confirm the diagnosis of NIVL, and stenting should be carried out only after such a diagnosis is confirmed. Antithrombotic therapy in the NIVL setting can be very selective, and lifelong follow-up is recommended.

## Figures and Tables

**Table 1:** Characteristics of studies considered. (NIVL: Non-thrombotic iliac vein lesion, QoL: Quality of life, IVUS: Intravascular ultrasound, CIVIQ: Chronic venous insufficiency questionnaire, CTV: Computed tomographic venography, CF: Common femoral, VAS: Visual Analog Pain score, VCSS: Venous clinical severity score, rVCSS: Revised VCSS, TAUS: Trans abdominal duplex ultrasound, EVLA: Endovenous laser ablation, R—Retrospective study, RCT—Randomized control trial).

Author-Year	Type of Study	‘n’ Limbs	NIVL (%)	Comparator	Diagnosis	Stent Types	Follow Up	Symptom Improvement	Signs	Score Improvement	Qol
Neglen 2007, [15]	R	982	53	None	Venography and IVUS	Wallstent; Nitinol	Mean—22 months (94%)	Grade of swelling improvement (1.7 to 0.8 *p* < 0.0001) VAS pain score improvement (3.7 to 0.8 *p* < 0.0001)	Recurrence free ulcer healing rate—63%		CIVIQ—20 score improvement (*p* < 0.001)
Ye 2012, [21]	R	224	100	None	CTV or CF vengraphy	Wallstent	Mean—50 (±36) months	Grade of swelling improvement (1.7 to 0.6 *p* < 0.01) VAS pain score improvement (4.3 to 0.4 *p* < 0.01)	48 month recurrence free ulcer healing rate—82.3%		CIVIQ score improvement (*p* < 0.001)
Jayaraj 2021, [16]	R	545	19	None	Venography and IVUS	Wallstent + Z stent	Median—26 months	Grade of swelling improvement (3 to 1 *p* < 0.0001) VAS pain score improvement (5 to 2 *p* < 0.0001)	Recurrence free ulcer healing rate—66%	VCSS improvement post stenting 6 to 4 (*p* < 0001)	Global CIVIQ—20 score improvement 60 to 36 (*p* < 0.0001)
Yang 2021, [22]	R	157	100	EVLA of incompetent superficial and/or perforator vein(s) with sclerotherapy/phlebectomy of varicose veins	CF venography	Wallstent	Median—24 months	Stenting + EVLA vs. EVLA: Mean VAS pain score improvement 2.3 vs. 1.1 (*p* = 0.01)	Stenting + EVLA vs. EVLA: Ulcer healing 82.8% vs. 68.8% (*p* = 0.03)	Stenting + EVLA vs. EVLA: Mean VCSS post stenting 8.3 vs. 11.7 (*p* = 0.01)	
Hong 2022, [23]	RCT	246	100	Venastent vs. Zilver Vena	Venography	Venastent or Zilver Vena	12 months		Ulcer healing rate (91% vs. 83% *p* = 0.99)	rVCSS improvement at 12 months (4.4 vs. 5)	
Ortega 2025, [24]	R	219	70	None	Venography + TAUS	Zilver Vena	Mean—41 (±22) months			Mean VCSS pain score improvement 6.9 to 1.4 (NIVL limbs)	

## Data Availability

No new data were created or analyzed in this study. Data sharing is not applicable to this article.
